# Author Correction: Densely vascularized thick 3D tissue shows enhanced protein secretion constructed with intermittent positive pressure

**DOI:** 10.1038/s42003-025-07809-2

**Published:** 2025-03-07

**Authors:** Misako Katsuura, Jun Homma, Yuhei Higashi, Hidekazu Sekine, Tatsuya Shimizu

**Affiliations:** 1 Institute of Advanced Biomedical Engineering and Science, TWIns, Tokyo Women’s Medical University, Tokyo, Japan; 2 Department of Pediatrics, Tokyo Women’s Medical University School of Medicine, Tokyo, Japan; 3 Tokaihit Co. Ltd, Shizuoka, Japan

**Keywords:** Biotechnology, Tissue engineering, Biological techniques

Correction to: *Communications Biology* 10.1038/s42003-025-07627-6, published online 08 February 2025

In the version of the article initially published, the Supplementary information was incomplete and omitted the gating strategy for flow cytometry. The corrected Supplementary information is now available online.

