# Supplementary material for: Densely vascularized thick 3D tissue shows enhanced protein secretion constructed with intermittent positive pressure
Source: Commun Biol. 2025 Feb 8;8:201. doi: 10.1038/s42003-025-07627-6 (PMC11807115; doi:10.1038/s42003-025-07627-6)
Supplement: Supplementary file 1 — Supplementary Information [file 42003_2025_7627_MOESM1_ESM.pdf]

Supplementary information

Method

Preparation of human adipose-derived stem cells (hASCs) in a planar environment and a cell sheet environment for RNA sequencing

For the hASCs in planar environment, 5.0×10^5 cells of hASCs were seeded onto a 12-well plate and cultured with basal medium for 2 days. For the hASC cell sheet environment, small-size cell sheets were prepared with 5.0×10^5 cells of hASCs. For constructing the cell sheet, hASCs were cultured with basal medium with 40 μg/ml ASA for 1 day. Then, the harvested hASC cell sheet was re-attached on a 35 mm cell culture dish for 1 day (Supplementary Fig. 1a).

Measurement of dissolved oxygen at the bottom of the culture dish and pH of the medium in the absence of a cell sheet during intermittent positive pressurization

Air saturation at the bottom of the dish was measured in the absence of a cell sheet using a multi-channel oxygen meter (OXY-4 mini; PreSens Precision Sensing GmbH, Regenburg, Germany) and an oxygen sensor tip (PreSens Precision Sensing

GmbH) (Supplementary Fig. 3a). Pressurization was initiated for the IPP (+) group, and air saturation was measured for both the IPP (-) and IPP (+) cultured dishes to assess dissolved oxygen levels in the medium. The pH of the medium was measured using a COMPACT pH METER (LAQUAtwin-pH-11, HORIBA, Kyoto, Japan) (Supplementary Fig. 3c).

Transplantation of cell sheets into same athymic rats

Seven male athymic rats (F344/NJcl-rnu/rnu; 220-290 g; 8–12 weeks of age; CLEA Japan, Tokyo, Japan) were used for the experiment shown in Supplementary Figure 4. In each rat, cell sheets of both IPP (-) and IPP (+) were transplanted into the right and left superficial gluteal muscles, and blood perfusion imaging was acquired 2 days later.

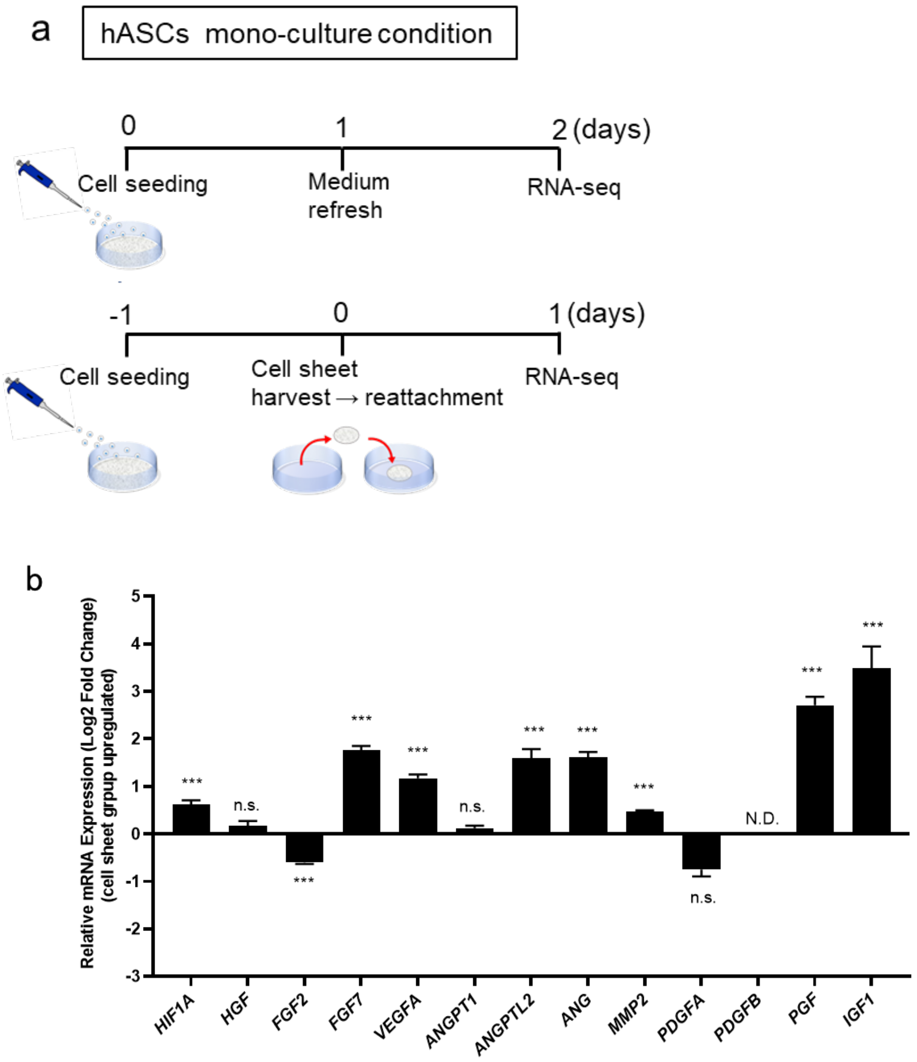

Supplementary Figure 1: Comparison of the RNA expression related to angiogenesis factor between hASCs planar culture and hASC sheet.

(a) Diagram of the mono-culture hASCs experiment. (b) To determine the relative mRNA expression values of hASCs cell sheet on the next day after reattachment to the culture dish, we used the mRNA expression values of hASCs cultured under planar conditions on day 2 as a reference (n = 3, independent samples, *** $p <$ 0.001).

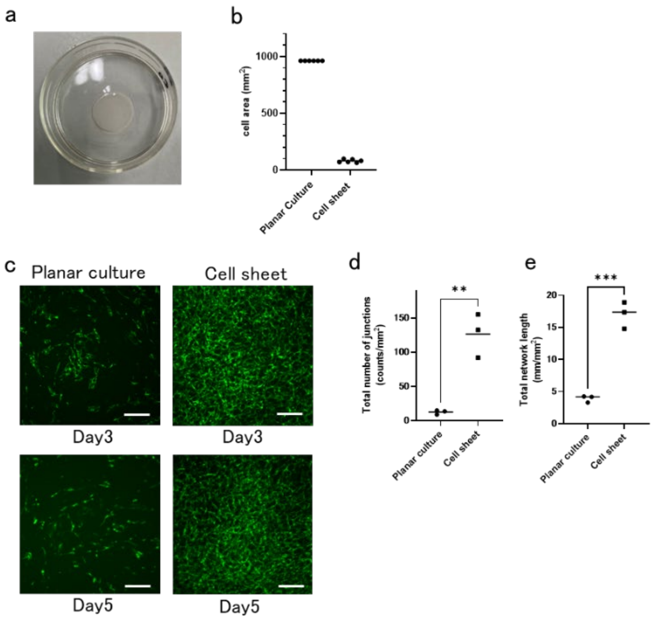

Supplementary Figure 2: Comparison of vascular network formation between planar co-cultured condition and co-cultured cell sheet condition.

(a) Representative image of a standard-sized cell sheet harvested from a 35 mm dish and reattached to a 35 mm dish. (b) The comparison of the cell area between the confluent cell state in a 35 mm dish and the cell sheet harvested from the same dish. (c) Representative images of GFP-HUVECs (*green*) in a planar co-cultured condition (*left*) and a co-cultured cell sheet condition (*right*) on day 3 and day 5. Scale bar: 500 µm. (d) Total number of endothelial network junctions on day 3 (n = 3, ** $p < 0.01$, Cohen's d = 5.55). (e) Total length of the endothelial network on day 3 (n = 3, ** $p < 0.01$, Cohen's d = 9.79). (d, e) Plots in the graph shows the value of each experiment and the bar shows the average. N = 3, representing 3 samples, with

the endothelial vascular network evaluated in 3 randomly selected fields of view per sample, and the analysis based on the mean of these fields.

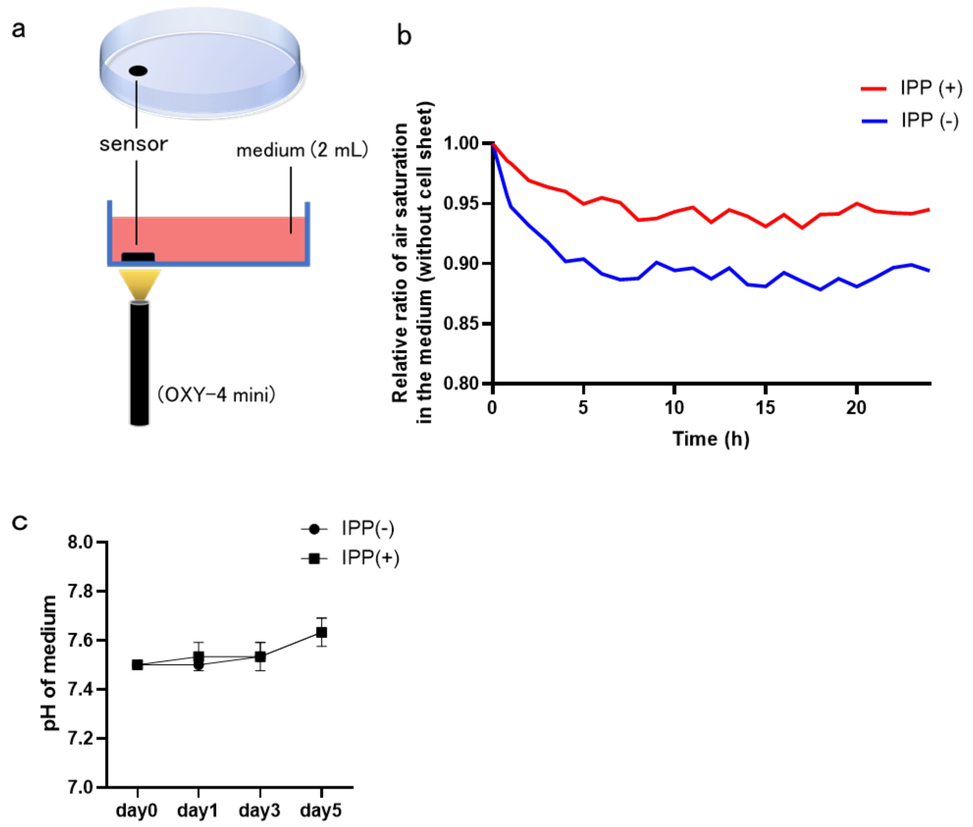

Supplementary Figure 3: Comparison of air saturation at the bottom of the dish and pH of the basal medium applied intermittent positive pressure and non-pressurization without cells.

(a) Schemic diagram for the measurement of air saturation at the bottom of the dish without a cell sheet. (b) Representative data showing relative changes in air saturation at the bottom of a culture dish in the absence of a cell sheet. (c) The daily changes in pH of the basal medium under IPP (-) / IPP (+) conditions (n = 3, independent samples).

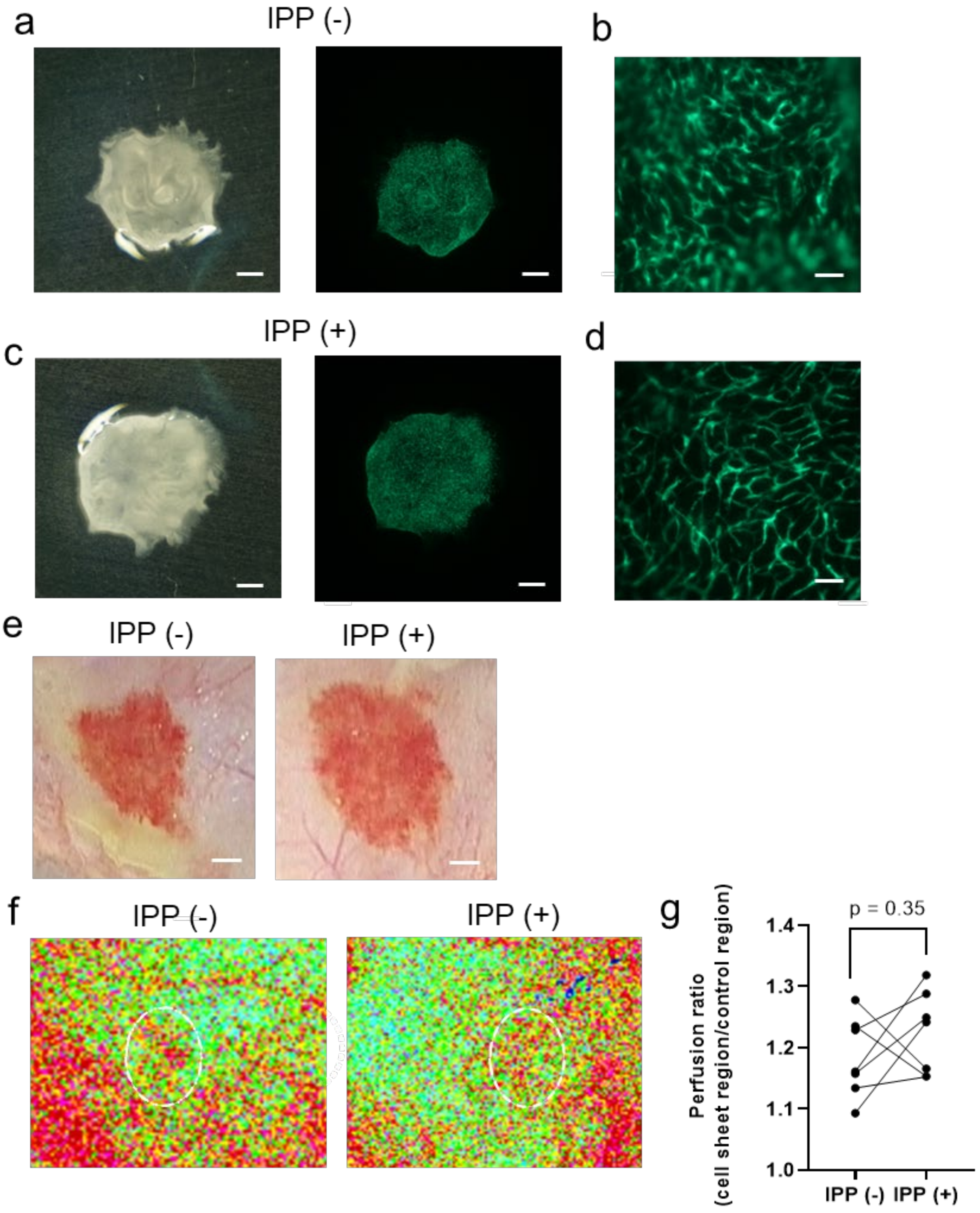
a
IPP (-)
b
c
IPP (+)
d
e
IPP (-)
IPP (+)
f
IPP (-)
IPP (+)
g
p = 0.35
1.4
1.3
1.2
1.1
1.0
Perfusion ratio
(cell sheet region/control region)
IPP (-)
IPP (+)

Supplementary Figure 4: Comparison blood perfusion of intermittently positive pressurized and non-pressurized cell sheets transplanted in the same rats.

(a) Representative stereomicroscope images of a co-cultured cell sheet cultured under IPP (-). The cell sheets were detached from the dish on day 5. Representative stereomicroscope image (*left*) and fluorescence stereoscope image (*right*) of a co-cultured cell sheet. Green indicates GFP-HUVECs. (b) A representative magnified fluorescence stereomicroscope image of (a). (c) Representative stereomicroscope images of a co-cultured cell sheet cultured under IPP (-). The cell sheets were detached from the dish on day 5. A representative stereomicroscope image (*left*) and fluorescence stereoscope image (*right*). Green indicates GFP-HUVECs. (d) Representative magnified fluorescence stereomicroscope image of (c). (e) Representative stereomicroscope images of a transplanted cell sheet of each condition. The image was captured 2 days after transplantation. (f) Representative images of perfusion signals of a transplanted cell sheet of each condition 2 days after transplantation. The dotted circle indicates the region of the transplanted cell sheet. (g) Perfusion ratio of transplanted cell sheets on 2 days after transplantation. (a, c, e) Scale bar: 1 mm. (b, d) Scale bar: 100 μm.

	IPP (-)			IPP (+)			Log_2 fold change	Fold change	p-value	Cohen's d
Gene Name	1	2	3	1	2	3				
NOTCH1	106.26	71.53353	93.09858	127.5521	123.3178	151.7819	0.572365907	1.486960071	5.94E-06	2.663713
NOTCH2	450.5611	519.0264	461.4898	414.3153	450.3178	422.6784	-0.152463332	-1.111465626	0.130209	1.639242
NOTCH3	423.3	380.6411	456.6655	510.7316	634.8149	669.626	0.526247475	1.44017834	4.22E-06	2.848332
NOTCH4	47.79381	36.69224	34.48856	45.39546	53.18547	50.83905	0.332598674	1.259279627	0.010368	1.756024
PIEZO1	225.5125	210.7898	230.2317	218.343	230.0085	236.7587	0.041010303	1.028834055	0.671153	0.635899
PIEZO2	1.160044	0.871032	1.026445	2.093162	1.492177	1.890709	0.822779525	-1.544139555	0.062494	3.36972
AGTR1	16.93664	18.61832	22.68444	13.8672	11.61767	12.49968	-0.626803145	-1.544139555	0.000168	3.016323
TRPA1	31.08918	83.94574	28.53518	26.94947	36.02543	21.42803	-0.766016679	-1.70056798	0.005183	0.868247
P2RX4	7.076268	5.552831	6.877183	7.456891	8.633312	9.558582	0.407156249	1.32606937	0.047023	2.162052
CDH5	97.21168	94.0715	125.8422	153.1933	216.2591	204.1965	0.855264112	1.809089912	9.17E-11	3.200306
KDR	84.3352	75.12654	89.40338	100.9951	137.3869	136.6562	0.592906834	1.508282672	7.58E-07	2.699566
NOS3	21.2288	17.09401	24.83997	34.40636	46.2575	48.21307	1.02997484	2.041988639	4.13E-12	3.681464
KLF2	12.99249	13.93652	20.32362	15.30625	19.50489	24.26409	0.32322216	1.251121725	0.093408	0.928764
NFKB1	54.98608	59.88348	53.4778	57.56197	48.2826	52.93984	-0.088929371	-1.063580603	0.39542	0.788371
AQP1_1	0.348013	0	0	0	0	0	-3.174732037	-9.030037863	0.222092	0.816569
ADRA1A	0	0	0	0	0	0.105039	1.902806554	3.739399354	0.471994	0.816484
HTR2A	5.336202	9.254719	6.569249	3.270566	8.84648	6.407401	-0.183628725	-1.135736947	0.528591	0.361038
ITGA5	845.208	897.5989	969.9907	900.5831	1110.82	1046.717	0.173020492	1.127416426	0.102621	1.300929
MYC	39.6735	28.96183	43.21334	49.32014	50.20111	56.8263	0.483480559	1.398112603	0.000357	2.473921
CDK1	2.552097	0.871032	3.079336	3.270566	1.492177	2.836063	0.213743693	1.159693607	0.615716	0.349263
CDK2	13.80452	14.91643	16.8337	17.9227	16.20078	15.86094	0.122030084	1.088265134	0.436906	1.105219
CDK6	143.9615	130.7637	156.7382	159.2112	181.0864	190.6465	0.300218112	1.231330557	0.004226	2.265801
CCNA1	0.812031	0.217758	1.026445	1.439049	1.598762	2.415905	1.400375285	2.639702393	0.007574	2.387256
CCND1	258.1098	282.5411	326.9228	323.2628	389.4583	389.486	0.345478135	1.270572003	0.002066	0.016827
CCND2	319.0121	394.1421	330.0021	317.7682	385.3015	347.8904	0.011112947	1.007732651	0.919839	0.069625
CCNB1	6.148233	2.721976	6.36396	5.887019	4.6897	6.827559	0.191436616	1.141900239	0.519116	0.443399
COL1A1	54155.03	45598	54710.56	61008.1	58484.4	58247.27	0.202498	1.150689028	0.04902	2.057632
COL1A2	32014.43	28368.11	31790.55	35144.85	33121.12	33152.32	0.137893519	1.100297397	0.163588	1.854813
COL4A1	922.235	948.5542	976.56	1115.525	1608.674	1326.122	0.508523892	1.422593909	1.43E-05	2.278071
COL4A2	686.63	712.6134	726.6206	792.9161	1143.647	947.4551	0.440186326	1.356779546	0.000161	2.019828
COL17A1	0.232009	0.653274	0.410578	1.046581	0.959257	1.260472	1.299698319	2.461773994	0.024166	3.543217
ITGA6	94.42758	91.24064	118.3491	158.9495	249.5134	245.372	1.10495302	2.150918737	6.46E-15	3.097578
LAMA4	721.5473	947.2477	741.9146	798.8031	1016.919	911.4266	0.178045557	1.131350188	0.13271	0.899797
LAMA5	38.86147	35.82121	45.47152	57.1695	77.80639	71.63685	0.783166233	1.720903539	2.27E-10	3.487813
LAMB1	1271.408	1524.524	1340.127	1245.17	1370.778	1370.344	-0.053098636	-1.037490867	0.607922	0.472079
LAMC1	1472.096	1668.136	1515.341	1538.736	1805.748	1642.291	0.099271596	1.07123247	0.334671	0.921108
HIF1A	1185.565	1958.734	1328.015	1015.969	1091.741	1005.962	-0.522400039	-1.436342733	0.000143	1.54649
EPAS1	319.8241	448.0373	344.5777	395.0844	475.7914	406.8175	0.199894522	1.148614375	0.099386	0.96423
HIF3A	3.712141	2.613097	2.05289	3.139744	2.451434	1.470551	-0.257344542	-1.195276624	0.492276	0.521466
ARNT	66.93454	74.7999	73.39083	60.70171	63.63071	63.12866	-0.19832268	-1.14736362	0.038786	2.912977
ARNT2	11.02042	9.363598	7.800984	8.241827	4.796285	6.302362	-0.552499696	-1.466624654	0.01231	1.765735
HGF	11.83245	17.96504	9.956519	8.37265	6.821383	8.928346	-0.720029607	-1.647215838	0.000951	1.70236
FGF2	154.4019	182.8079	146.679	178.1805	176.5033	145.6896	0.047807752	1.033692981	0.668031	0.294429
FGF7	120.1806	314.5516	154.3774	85.55802	82.70926	80.5652	-1.24331438	-2.367417875	6.69E-09	1.545401
VEGFA	332.7006	362.1317	279.3984	390.6364	441.3648	299.0471	0.215318381	1.160960093	0.094543	0.886004
ANGPT1	113.1043	219.7179	139.9045	82.94156	83.98827	87.07764	-0.896049382	-1.860963013	3.33E-08	1.857706
ANGPTL2	114.9604	122.38	116.5015	115.778	100.722	104.1991	-0.146378037	-1.106787333	0.140382	1.774964
ANG	1.508057	1.19767	1.231734	2.354808	0.959257	1.68063	0.307378843	1.237457385	0.495268	0.693772
MMP2	2006.296	2437.257	2030.103	1787.43	1769.296	1665.504	-0.309962472	-1.239675453	0.003211	2.350258
PDGFA	10.4404	6.968259	14.26759	15.82954	19.61147	19.01213	0.780540202	1.717773955	0.000126	2.569431
PDGFB	8.584325	6.314985	9.340651	12.16651	21.52999	13.23496	0.954714798	1.93819644	2.65E-05	1.994969
PGF	83.98718	68.92044	86.63198	96.28547	118.3084	121.5305	0.490072069	1.404515035	1.35E-05	2.720601
IGF1	5.220198	5.770589	4.31107	4.578793	2.664603	2.625984	-0.660171054	-1.580269978	0.02292	1.914225

Supplementary Table 1: Raw count per million (CPM) data and statistical analysis for RNA sequencing in figure 6.

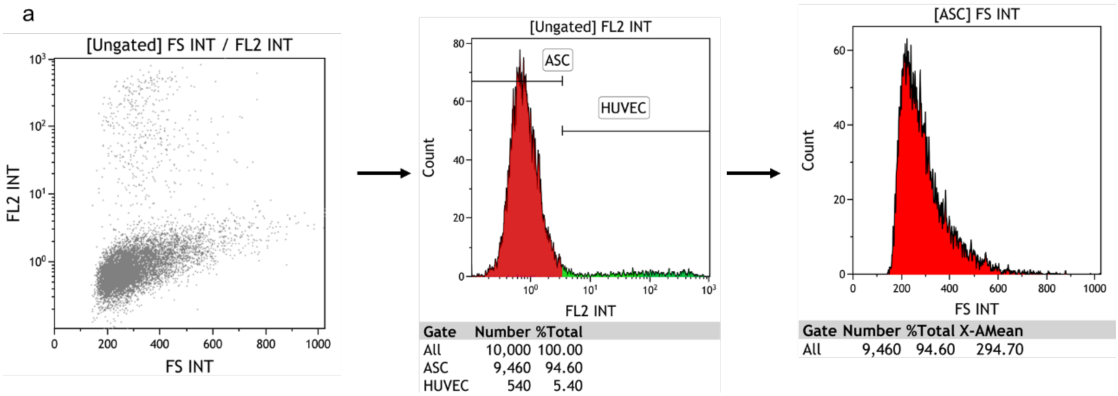

Supplementary Information: Flow cytometry strategy for cell size assessment in co-cultured cell sheets

a. A representative flow cytometry analysis of the co-cultured populations of hASCs and GFP-HUVECs.

Flow cytometry procedure and analysis of co-cultured cell sheets:

Co-cultured cell sheets containing hASCs and GFP-HUVECs were dissociated using trypsin-EDTA solution (FUJIFILM Wako Pure Chemical Corporation) and 0.5 mg/ml type II collagenase (Worthington Biomedical Corporation) to obtain single-cell suspensions. These suspensions were then analyzed using a Gallios flow cytometer (Beckman Coulter, Inc., Tokyo, Japan).

For the initial analysis, cells were plotted on an FL2 INT vs. FS INT scatter plot. FL2 INT represents the green fluorescence intensity of GFP-HUVECs, while FS INT (forward scatter intensity) reflects the cell size for both hASCs and GFP-HUVECs. It is important to note that debris removal and singlet exclusion were not performed, meaning that debris

and other non-cellular particles may have been included in the analysis.

To identify cell populations more clearly, further analysis was performed based on FSC (forward scatter) values, which indicate cell size. GFP-HUVECs were distinguished by their high fluorescence in the FL2 channel, while hASCs were identified as cells with low or no fluorescence in the FL2 channel.

Data analysis was performed using Kaluza Analysis 2.1 software (Beckman Coulter, Inc.) to quantify the percentage of each cell population based on their FSC values, enabling the differentiation of GFP-HUVECs and hASCs. As debris removal and singlet exclusion were not applied, all dissociated cells—including debris and doublets—were included in the analysis.
