# Supplementary material for: Densely vascularized thick 3D tissue shows enhanced protein secretion constructed with intermittent positive pressure
Source: Commun Biol. 2025 Feb 8;8:201. doi: 10.1038/s42003-025-07627-6 (PMC11807115; doi:10.1038/s42003-025-07627-6)
Supplement: Supplementary file 3 — Description of Additional Supplementary File [file 42003_2025_7627_MOESM3_ESM.pdf]

Description Of Additional Supplementary Data

File name: Supplementary data 1.

Description: Source data for each analysis.
