# Supplementary material for: Densely vascularized thick 3D tissue shows enhanced protein secretion constructed with intermittent positive pressure
Source: Commun Biol. 2025 Feb 8;8:201. doi: 10.1038/s42003-025-07627-6 (PMC11807115; doi:10.1038/s42003-025-07627-6)
Supplement: Supplementary file 4 — reporting summary [file 42003_2025_7627_MOESM4_ESM.pdf]

Corresponding author(s): Jun Homma and Sekine Hidekazu

Last updated by author(s): Jan 16, 2025

Reporting Summary

Nature Portfolio wishes to improve the reproducibility of the work that we publish. This form provides structure for consistency and transparency in reporting. For further information on Nature Portfolio policies, see our Editorial Policies and the Editorial Policy Checklist.

Statistics

For all statistical analyses, confirm that the following items are present in the figure legend, table legend, main text, or Methods section.

n/a	Confirmed	
☐	☒	The exact sample size (*n*) for each experimental group/condition, given as a discrete number and unit of measurement
☐	☒	A statement on whether measurements were taken from distinct samples or whether the same sample was measured repeatedly
☐	☒	The statistical test(s) used AND whether they are one- or two-sided<br>*Only common tests should be described solely by name; describe more complex techniques in the Methods section.*
☒	☐	A description of all covariates tested
☐	☒	A description of any assumptions or corrections, such as tests of normality and adjustment for multiple comparisons
☐	☒	A full description of the statistical parameters including central tendency (e.g. means) or other basic estimates (e.g. regression coefficient) AND variation (e.g. standard deviation) or associated estimates of uncertainty (e.g. confidence intervals)
☐	☒	For null hypothesis testing, the test statistic (e.g. *F*, *t*, *r*) with confidence intervals, effect sizes, degrees of freedom and *P* value noted<br>*Give P values as exact values whenever suitable.*
☒	☐	For Bayesian analysis, information on the choice of priors and Markov chain Monte Carlo settings
☒	☐	For hierarchical and complex designs, identification of the appropriate level for tests and full reporting of outcomes
☒	☐	Estimates of effect sizes (e.g. Cohen's *d*, Pearson's *r*), indicating how they were calculated

Our web collection on statistics for biologists contains articles on many of the points above.

Software and code

Policy information about availability of computer code

Data collection	Angio Tool v0.5a software (National Institute of Health, Maryland, USA)
Data analysis	GraphPad Prism Version 9.5.1 (733)

For manuscripts utilizing custom algorithms or software that are central to the research but not yet described in published literature, software must be made available to editors and reviewers. We strongly encourage code deposition in a community repository (e.g. GitHub). See the Nature Portfolio guidelines for submitting code & software for further information.

Data

Policy information about availability of data

All manuscripts must include a data availability statement. This statement should provide the following information, where applicable:

- Accession codes, unique identifiers, or web links for publicly available datasets
- A description of any restrictions on data availability
- For clinical datasets or third party data, please ensure that the statement adheres to our policy

All data supporting the findings of this study are included within the article (and its supplementary files). Additional data are available from the corresponding author upon reasonable request. All RNA-seq data have been uploaded to the Sequence Read Archive (SRA) under the project accession number PRJNA1163514. Source data for each analysis can be obtained in Supplementary Data 1.

Research involving human participants, their data, or biological material

Policy information about studies with human participants or human data. See also policy information about sex, gender (identity/presentation), and sexual orientation and race, ethnicity and racism.

Reporting on sex and gender	This study does not include data regarding human sexuality or gender.
Reporting on race, ethnicity, or other socially relevant groupings	This study does not include data regarding race, ethnicity, or other socially relevant, groupings.
Population characteristics	This study does not include information from human participants or human data.
Recruitment	This study does not include information from human participants or human data.
Ethics oversight	This study does not include information from human participants or human data.

Note that full information on the approval of the study protocol must also be provided in the manuscript.

Field-specific reporting

Please select the one below that is the best fit for your research. If you are not sure, read the appropriate sections before making your selection.

☒ Life sciences ☐ Behavioural & social sciences ☐ Ecological, evolutionary & environmental sciences

For a reference copy of the document with all sections, see nature.com/documents/nr-reporting-summary-flat.pdf

Life sciences study design

All studies must disclose on these points even when the disclosure is negative.

Sample size	The sample size calculation was not performed. The sample size was set to n=3 or more, depending on the experimental context. The selection of sample size was based on considerations of data variability and available experimental resources.
Data exclusions	For the analysis of figure 8j (Serum GLuc density), n=1 was excluded because the rat's weight differed by over 6% between the control and IPP(+) groups.
Replication	To verify the reproducibility of the experimental results, the same experiments were conducted multiple times. The same researchers followed the same protocol for each experiment to ensure reproducibility.
Randomization	In this experiment, samples for the IPP(-) and IPP(+) groups were randomly assigned. The specimens before the pressure application were from co-cultured cells with the same cellular composition, and the protocol naturally ensured random allocation.
Blinding	Blinding could not be implemented in this experiment because the experimental procedures were primarily conducted by the first author. Additionally, since the experiment aimed to examine differences between the IPP(-) and IPP(+) groups, multiple individuals were involved in verifying the samples to prevent mix-ups between IPP(-) and IPP(+) samples. As a result, there were not enough personnel available to implement blinding.

Reporting for specific materials, systems and methods

We require information from authors about some types of materials, experimental systems and methods used in many studies. Here, indicate whether each material, system or method listed is relevant to your study. If you are not sure if a list item applies to your research, read the appropriate section before selecting a response.

Materials & experimental systems

n/a	Involved in the study	
☐	☒	Antibodies
☒	☐	Eukaryotic cell lines
☒	☐	Palaeontology and archaeology
☐	☒	Animals and other organisms
☒	☐	Clinical data
☒	☐	Dual use research of concern
☒	☐	Plants

Methods

n/a	Involved in the study	
☒	☐	ChIP-seq
☐	☒	Flow cytometry
☒	☐	MRI-based neuroimaging

Antibodies

Antibodies used	Antibodies used in the study are shown in Table1 in the manuscript.
Validation	◆Anti-RFP (RABBIT) Antibody(URL:https://www.rockland.com/categories/primary-antibodies/rfp-antibody-pre-adsorbed-600-401-379/?srsltid=AfmBOoop1-xZ6OInwwPP-giKZDqrHVogz2lMGzG7i5jVLWvcC4ngM2OF):This product was prepared from monospecific antiserum by immunoaffinity chromatography using Red Fluorescent Protein (Discosoma) coupled to agarose beads followed by solid phase adsorption(s) to remove any unwanted reactivities. Expect reactivity against RFP and its variants: mCherry, tdTomato, mBanana, mOrange, mPlum, mOrange and mStrawberry. Assay by immunoelectrophoresis resulted in a single precipitin arc against anti-Rabbit Serum and purified and partially purified Red Fluorescent Protein (Discosoma). No reaction was observed against Human, Mouse or Rat serum proteins.<br>◆CoraLite® 594-conjugated INS Monoclonal antibody(URL:https://www.ptglab.com/products/Insulin-Antibody-CL594-66198.htm#tested-applications):Tested applications; Positive IF-P detected in mouse pancreas tissue. CL594-66198 targets INS in IF-P applications and shows reactivity with human, mouse, rat samples.<br>◆CD31 (PECAM-1) (89C2)Mouse mAb (Alexa Fluor® 488 Conjugate)(URL:https://www.cellsignal.jp/products/antibody-conjugates/cd31-pecam-1-89c2-mouse-mab-alexa-fluor-488-conjugate/42777):CD31 (PECAM-1) (89C2) Mouse mAb (Alexa Fluor® 488 Conjugate) detects endogenous levels of total CD31 protein. It does not cross-react with other related proteins.<br>◆Hoecst 33258: (Batch no. BCBM1002V URL:https://www.sigmaaldrich.com/JP/ja/product/sigma/94403#product-documentation, https://www.sigmaaldrich.com/certificates/Graphics/COfAInfo/fluka/pdf/PDF498659.pdf)

Animals and other research organisms

Policy information about studies involving animals; ARRIVE guidelines recommended for reporting animal research, and Sex and Gender in Research

Laboratory animals	Athymic rats (F344/NJcl-rnu/rnu; 180-290 g; CLEA Japan, Tokyo, Japan), 8-12w.
Wild animals	N/A
Reporting on sex	This experiment was not designed to evaluate sex differences, so it was conducted using only male rats. Differences in results based on sex were not assessed in this experiment.
Field-collected samples	Field-collected samples were not included in this experiment.
Ethics oversight	All animal experiments were performed following the guidelines of the Ethics Committee for Animal Experimentation of Tokyo Women's Medical University and in compliance with the Legislation and Regulation on the use of animals in biological research with the ARRIVE guidelines. All experimental protocols were approved by the Ethics Committee for Animal Experimentation of Tokyo Women's Medical University (Approval Number AE23-122, AE24-021).

Note that full information on the approval of the study protocol must also be provided in the manuscript.

Plants

Seed stocks	No plants were used.
Novel plant genotypes	No plants were used.
Authentication	No plants were used.

Flow Cytometry

Plots

Confirm that:

☒ The axis labels state the marker and fluorochrome used (e.g. CD4-FITC).

☒ The axis scales are clearly visible. Include numbers along axes only for bottom left plot of group (a 'group' is an analysis of identical markers).

☒ All plots are contour plots with outliers or pseudocolor plots.

☒ A numerical value for number of cells or percentage (with statistics) is provided.

Methodology

Sample preparation	The co-cultured cell sheets were enzymatically dissociated into single-cell suspensions using trypsin-EDTA solution (FUJIFILM Wako Pure Chemical Corporation) and 0.5 mg/ml type II collagenase (Worthington Biomedical Corporation).
Instrument	Flow cytometry analysis was performed using a Gallios cytometer (Beckman Coulter, Inc., Tokyo, Japan).
Software	The data were analyzed with Kaluza Analysis 2.1 software.
Cell population abundance	hASCs: Approximately 95% (based on FL2 analysis), GFP-HUVECs: Approximately 5% (based on FL2 analysis).
Gating strategy	In this study, debris removal and singlet cell exclusion were not performed, and all dissociated cells (including debris and doublets) were included in the analysis. The analysis was conducted using FSC values (cell size) to identify cell populations, and data were quantified using Kaluza Analysis 2.1 software.

☒ Tick this box to confirm that a figure exemplifying the gating strategy is provided in the Supplementary Information.
